# Antioxidative effects of caffeine in a hyperoxia-based rat model of bronchopulmonary dysplasia

**DOI:** 10.1186/s12931-019-1063-5

**Published:** 2019-05-10

**Authors:** Stefanie Endesfelder, Evelyn Strauß, Till Scheuer, Thomas Schmitz, Christoph Bührer

**Affiliations:** Department of Neonatology, Charité Universitätsmedizin Berlin, Augustenburger Platz 1, 13353 Berlin, Germany

**Keywords:** Caffeine, Hyperoxia (oxygen), Preterm, Animal model, Oxidative stress, Antioxidant

## Abstract

**Background:**

While additional oxygen supply is often required for the survival of very premature infants in intensive care, this also brings an increasing risk of progressive lung diseases and poor long-term lung outcomes. Caffeine is administered to neonates in neonatal intensive care for the prevention and treatment of apneas and has been shown to reduce BPD incidence and the need for mechanical ventilation, although it is still unclear whether this is due to a direct pulmonary action via antagonism of adenosine receptors and/or an indirect action. This experimental study aims to investigate the action of caffeine on the oxidative stress response in pulmonary tissue in a hyperoxia-based model of bronchopulmonary dysplasia in newborn rats.

**Methods:**

Newborn Wistar rats were exposed to 21% or 80% oxygen for 3 (P3) or 5 (P5) postnatal days with or without recovery on room air until postnatal day 15 (P15) and treated with vehicle or caffeine (10 mg/kg) every 48 h beginning on the day of birth. The lung tissue of the rat pups was examined for oxidative stress response at P3 and P5 immediately after oxygen exposure or after recovery in ambient air (P15) by immunohistological staining and analysis of lung homogenates by ELISA and qPCR.

**Results:**

Lungs of newborn rats, corresponding to the saccular stage of lung development and to the human lung developmental stage of preterms, showed increased rates of total glutathione and hydrogen peroxide, oxidative damage to DNA and lipids, and induction of second-phase mediators of antioxidative stress response (superoxide dismutase, heme oxygenase-1, and the Nrf2/Keap1 system) in response to hyperoxia. Caffeine reduced oxidative DNA damage and had a protective interference with the oxidative stress response.

**Conclusion:**

In addition to the pharmacological antagonism of adenosine receptors, caffeine appears to be a potent antioxidant and modulates the hyperoxia-induced pulmonary oxidative stress response and thus protective properties in the BPD-associated animal model. Free-radical-induced damage caused by oxidative stress seems to be a biological mechanism progress of newborn diseases. New aspects of antioxidative therapeutic strategies to passivate oxidative stress-related injury should be in focus of further investigations.

**Electronic supplementary material:**

The online version of this article (10.1186/s12931-019-1063-5) contains supplementary material, which is available to authorized users.

## Background

Preterm birth and administration of supplemental oxygen have a profound impact on pulmonary lung development [1–5]. Disruption of postnatal development is associated with pulmonary dysfunction, higher risk of respiratory diseases such as respiratory distress syndrome or bronchopulmonary dysplasia (BPD), and higher rates of lifelong morbidities [6–9]. The incidence of BPD correlates closely with the immaturity of the lung, such as poor oxygen detoxification enzymes and oxidative stress response [10, 11]. Besides the prematurity and the surfactant deficiency, the mechanical ventilation, the oxygen toxicity, and thus the effect of high oxygen concentrations with the resulting oxidative stress is an important cause of BPD [11–14]. Oxidative stress indicates an imbalance between reactive oxygen species (ROS) and corresponding antioxidant counterparts, resulting in toxic levels of ROS and damage to lipids, proteins, and DNA [15]. It is necessary to differentiate between physiological ROS for redox-sensitive signal transduction pathways and pathologically relevant ROS levels, which could affect vulnerable processes such as lung development [16], aggravated by inflammatory side effects [11]. Preterm infants are particularly exposed to the oxidative stress due to the transition from intrauterine hypoxia to extrauterine hyperoxia and have an increased risk of BPD. Besides avoiding premature birth, prenatal glucocorticoids, reducing oxygen content in ventilated air, gentle ventilation techniques, and surfactant therapy, antioxidative therapy strategies represent another possibility for preventing BPD. [17]. Existing therapies can reduce the rate of BPD but show minimal effects on BPD-typical arrest of lung development [18, 19]. New insights could be offered by antioxidant therapies if they reduce oxidative stress per se and thus could minimize the pathological consequences [20].

The methylxanthine caffeine is the standard and most frequently used medication for treatment of apnea due to prematurity. Caffeine acts mainly as a nonspecific inhibitor of adenosine receptors subtypes A1 and A2a and with a broad spectrum of pharmacokinetic activity [21]. In a large clinical trial, caffeine has been shown to reduce the rate of BPD in very preterm infants, shortening the duration time of mechanical ventilation and oxygen supplementation [22]. Caffeine appears to be most effective when started within the first few days of life [23–25].

In experimental animals, BPD-like lung damage can be precipitated by postnatal hyperoxia [26], with similar histological changes in hyperoxia-exposed rodent pups and human preterm infants [18, 27, 28]. These models can be used to study the effect of therapeutic interventions such as caffeine, which are not completely understood [29–31]. In addition to antagonism of adenosine receptors [32], anti-inflammatory effects, and the reduction of stress on the endoplasmic reticulum, anti-oxidant properties per se have also been discussed [33–36]. Therefore, this study aims to investigate the action of caffeine on the oxidative stress response in pulmonary tissue in a hyperoxia-based model of BPD in the newborn rat.

## Methods

### Oxygen exposure and drug administration

**Fig. 1:**
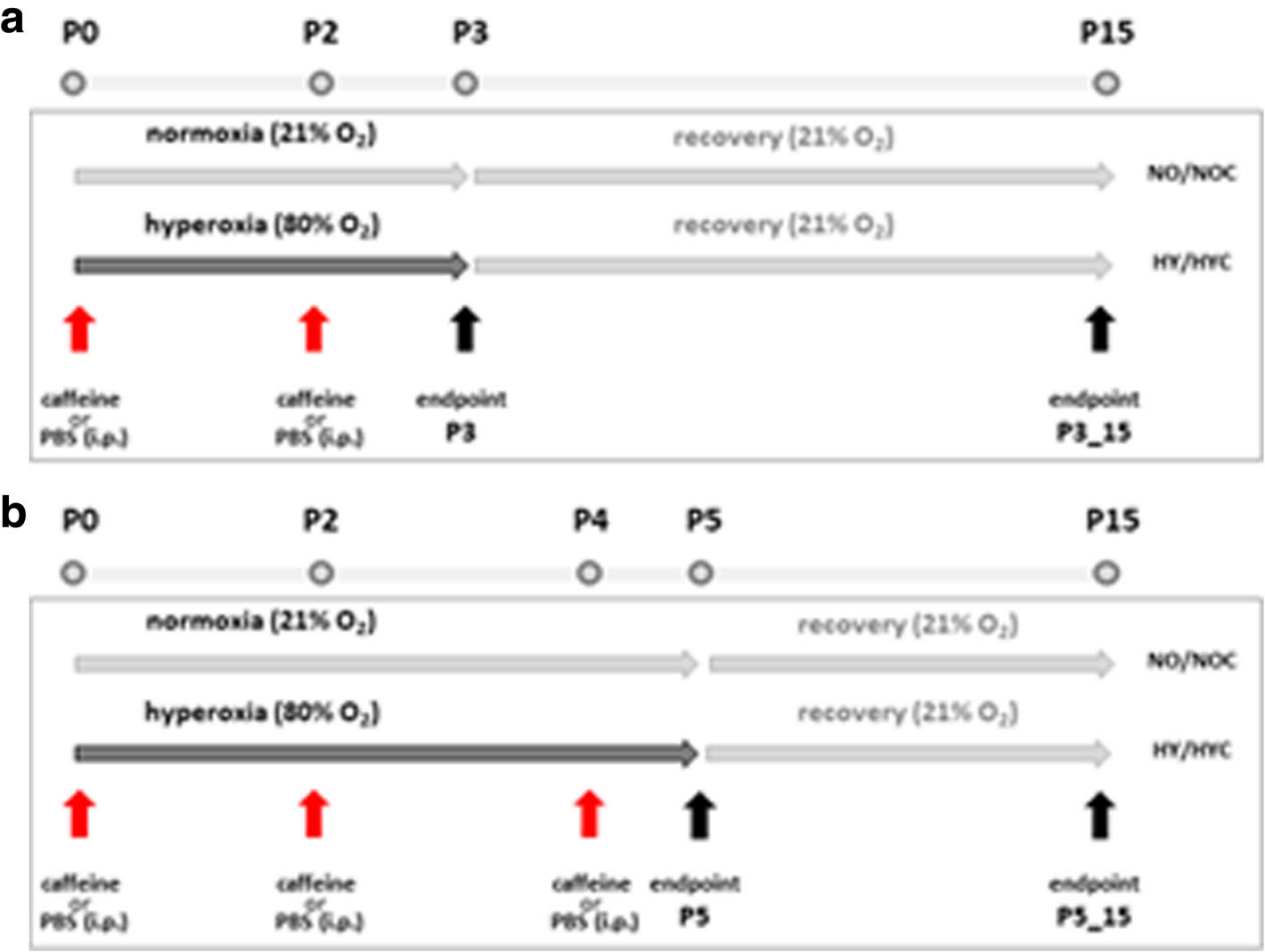
Time course of hyperoxic exposure and recovery of rat pups. The pups were divided into four groups with exposure to room air or hyperoxia occurring for either **a**) 3 postnatal days (P0-P3) or **b**) 5 postnatal days (P0-P5) with normoxia (NO) and vehicle application (PBS), normoxia with caffeine application (NOC), hyperoxia (HY) and vehicle application, and hyperoxia with caffeine (HYC). Rat pups received caffeine or vehicle injection intraperitoneally as a fixed portion of body weight (100 μl/10 g) every 48 h beginning on the day of birth (P0, red arrow). The experimental endpoints (black arrow) are directly after the oxygen exposure (P3 or P5) and after recovery in room air at P15 (P3_P15 or P5_P15)

Time-pregnant *Wistar* rat dams were obtained from the Department of Experimental Medicine (FEM, Charité – Universitätsmedizin Berlin, Germany). The adult rats were housed in individual cages under environment-controlled conditions with a constant 24 h light/dark cycle, ambient temperature, and relative humidity of 80% with *ad libitum *access to the same food and water. All procedures were approved by the local animal welfare authorities (LAGeSo, approval number G-0088/16) and followed institutional guidelines. The pups were pooled and randomized within 12 h of birth and returned to the dams. According to the experimental conditions (see Fig. 1), the newborn rats were randomly assigned to room air (normoxia) or oxygen-enriched atmosphere (hyperoxia) treatment. The pups in hyperoxia subgroups (HY) were reared with the dams in an atmosphere containing 80% oxygen (OxyCycler BioSpherix, Lacona, NY) from A) postnatal day (P)0 to P3 (*n = 7–8*) or B) P0 to P5 (*n = 6–8*); in parallel, the pups in normoxia (NO) groups were reared with the dams under room air conditions. To avoid oxygen toxicity in the nursing mothers, they were rotated between the oxygen treatment and normoxia litters every 24 h. The rats are divided into four groups, each for both exposure times with 1) normoxia (NO, control group): 21% oxygen application of vehicle (phosphate buffered saline, PBS), 2) normoxia with caffeine (NOC): 21% oxygen with caffeine (10 mg/kg, Sigma, Steinheim, Germany), 3) hyperoxia (HY): 80% oxygen with vehicle (PBS), and 4) hyperoxia with caffeine (HYC): 80% oxygen with caffeine (10 mg/kg). 10 mg/kg of pure caffeine is equivalent to 20 mg/kg caffeine citrate, which used clinically.

Rat pups received either drug or vehicle injection intraperitoneally (i.p.) as a fixed proportion of their body weight (100 μl/10 g) every 48 h beginning on the day of birth (P0). The administration of caffeine or vehicle took place for the pups with a total of three postnatal days of oxygen exposure (P0-P3) on the day of birth (P0) and on P2; for the rat pups with a total of five days of postnatal oxygen exposure (P0-P5) on the day of birth (P0) and on P2 and P4. The rat pups were first examined after the oxygen exposure (P3, P5). Further examinations occurred after recovery in room air at P15 (P3_P15, P5_P15). No pups died during hyperoxia.

### Plasma and tissue preparation

At the experimental endpoints (P3; P3_P15; P5; P5_P15), rat pups were anaesthetized with an i.p. injection of ketamine (100 mg/kg), xylazine (20 mg/kg), and acepromazine (3 mg/kg), blood samples were collected into heparin-coated centrifugation microtubes (Microvette CB300LH, Sarstedt International, Nümbrecht, Germany), and then transcardially perfused. The heart lung block was immediately removed and the lungs were snap-frozen in liquid nitrogen and stored at − 80 °C. The perfusion was carried out with PBS (pH 7.4) for the molecular analysis and for immunohistochemical analysis followed by perfusion with 4% paraformaldehyde (pH 7.4), the lungs were postfixed at 4 °C for 1 day, embedded in paraffin, and processed for histological staining. The blood samples were centrifuged at 4 °C, the plasma samples were separated and stored at − 80 °C. The animals were weighed at each time point of application (P0, P2 and/or P4) and at the experimental endpoints (P3/P5 and P15).

### Caffeine plasma level

The concentrations of caffeine in the plasma were determined externally using the LC-MS/MS analytical method (Institut für Veterinärmedizinische Diagnostik GmbH, Berlin, Germany).

### RNA extraction and quantitative real-time PCR

The gene expression analysis was performed as previously described [37]. The PCR products of *hypoxanthine-guanine phosphoribosyl-transferase* (*Hprt*), *Kelch-like ECH-associated protein 1* (*Keap1*), *NFE2-related factor 2* (*Nrf2*), and *superoxide dismutase* (*Sod*) *1*, *2*, and *3* were quantified in real time with the sequences (synthesized by BioTeZ, Berlin, Germany) summarized in Additional file 2: Table S1. PCR and detection were performed with qPCR BIO Mix Hi-ROX (NIPPON Genetics Europe, Düren, Germany) with *Hprt* used as an internal reference. The expression of target genes was analyzed with the StepOnePlus real-time PCR system (Applied Biosystems, Carlsbad, CA, USA) according to the 2^-ΔΔCT^ method [38].

### Protein extraction

Protein was extracted were determined using the Pierce BCA kit (Pierce/Thermo Scientific, Rockford, IL, USA) as described in [39].

### Oxidative stress marker ELISA assay

Concentrations of total glutathione (OxiSelect Total Glutathione Assay Kit, Cell Biolabs Inc., San Diego, CA, USA), lipid peroxidation (TBARS assay kit, Cayman Chemical, Ann Arbor, MI, USA), heme oxygenase (HO)-1 (Rat Heme Oxygenase-1 EIA Kit, Precoated, Takara Bio Europe/SAS, Saint-Germain-en-Laye, France), and hydrogen peroxide (H_2_O_2_; OxiSelect Hydrogen peroxide assay kit, Cell Biolabs Inc.) were measured in lung homogenates according to manufacturer’s instructions. Values were calculated from a standard curve and normalized to the amount of total protein. Superoxide dismutase (Sod) 1, 2, and 3 concentrations were measured using rat colorimetric ELISA kits (antibodies-online GmbH, Aachen, Germany).

### Immunohistochemistry

Ten-micrometer-thick paraffin lung sections were deparaffinized in xylene, rehydrated in ethanol, and subjected to hematoxylin and eosin (HE) staining or immunostaining.

The pulmonary morphometric changes were viewed by light microscopy. Statistical data on structural changes has not been compiled because other perfusion techniques must be used according to the required standard. The exemplary HE stains are only intended to support the statement about the effectiveness of oxygen toxicity as a damage model of the lung (Additional file 1: Figure S1).

Deparaffinized sections were immunostained with an anti-8-oxodG monoclonal antibody (clone 2E2; Trevigen, Gaithersburg, MD, USA) as previously described in [34]. Sections were analyzed blind using a Keyence compact fluorescent microscope BZ 9000 with BZ-II Viewer software and BZ-II Analyzer software (Keyence, Osaka, Japan). 8-oxodG positive cells were counted in 4 non-overlapping separate fields per animal and mean values of all images were used for statistical analysis.

### Statistical analyses

All data are expressed as the mean ± standard error of the mean (SEM). Groups were compared assuming a non-Gaussian distribution with the Mann Whitney test. A two-sided *p* value of < 0.05 was considered significant. All graphics and statistical analyses were performed using the GraphPad Prism 8.0 software (GraphPad Software, La Jolla, CA, USA).

## Results

### Caffeine alters postnatal weight gain

**Fig. 2:**
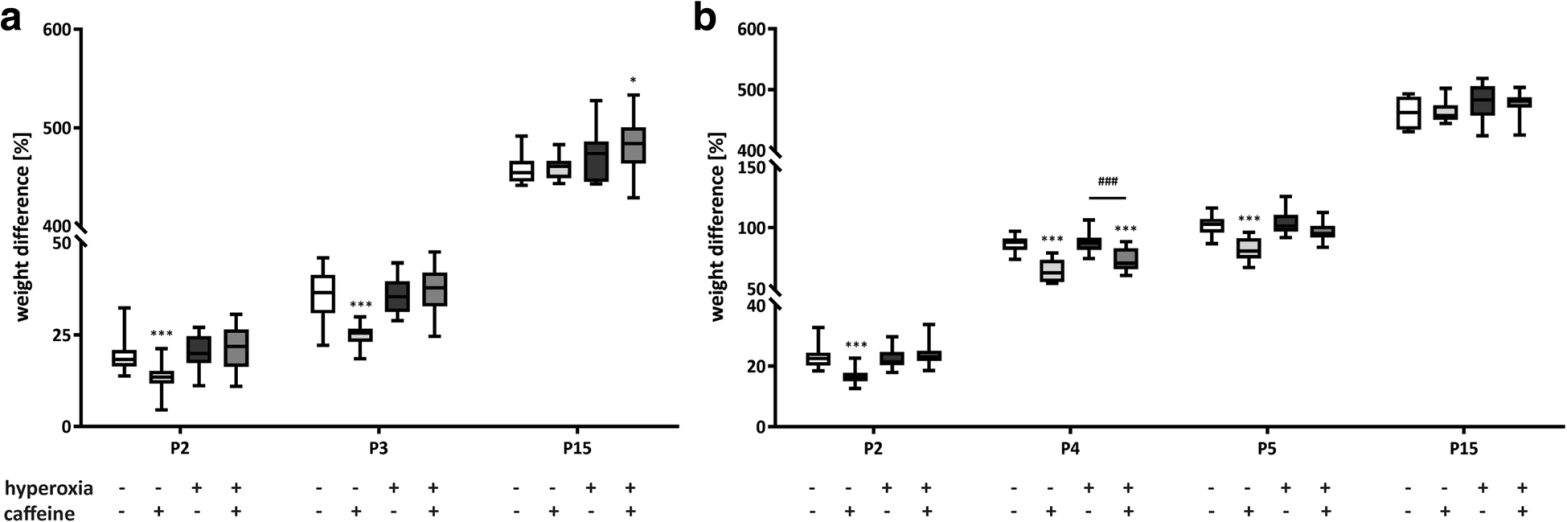
Weight gain of rat pups exposed to normoxia or hyperoxia and treated or not with caffeine for the examination groups **a**) oxygen exposure from P0 to P3 with monitored body weight on P2, P3, and P15, and **b**) oxygen exposure from P0 to P5 with monitored body weight on P2, P4, P5, and P15. Values are the mean of the difference in weight of all animals used for immunohistochemical and molecular biological experiments (*n* = 14–16). Data are expressed relative to the control group at each time point as mean ± SEM. *p < 0.05, **p < 0.01, and ***p < 0.001 versus control (NO); ###p < 0.001 versus hyperoxia group (HY) with Mann Whitney test

The effects of caffeine treatment on the weight gain of newborn rats were monitored for the different experimental groups at different time points. It was found that caffeine reduced weight gain in control rats (normoxia) at day 2 (P2), day 4 (P4), and day 5 (P5), while in hyperoxic pups, caffeine treatment inhibited weight gain only at P2 and P4 (Fig. 2 a and b). By age P15, no difference in body weight was found for any of the experimental groups, which indicating temporary effects of caffeine on early postnatal weight gain.

### Plasma caffeine level

Plasma caffeine levels were undetectable in the saline-treated pups at P3 and P5, as well in all groups after recovery at P3_P15 and P5_P15. The levels for caffeine-treated normoxic exposed rat pups were 9.4 ± 0.8 μg/ml at P3 and 10.4 ± 0.8 μg/ml at P5, and for caffeine-treated hyperoxic exposed rat pups 8.8 ± 0.5 μg/ml and 11.2 ± 0 μg/ml, respectively, without significant differences between groups. Validation of plasma caffeine levels showed that caffeine was sufficient to be within the therapeutically comparable range (5.5–23.7 mg/L) [21].

### Caffeine attenuates morphological changes

Hyperoxia for 3 or 5 days immediately after birth caused substantial alterations in lung morphology. After oxygen exposure, the lungs of rats pups contained fewer alveoli than those of control pups under normoxia conditions, with generalized enlargement of alveolar airspaces. These histological changes were even more pronounced after 5 days of exposure. After recovery under normoxic conditions to postnatal day 15, morphological changes were still detected in the oxygen-damaged lungs. Caffeine effectively counteracted these structural changes (Additional file 1: Figure S1).

### Caffeine reduces the oxidative stress-induced DNA damage

**Fig. 3:**
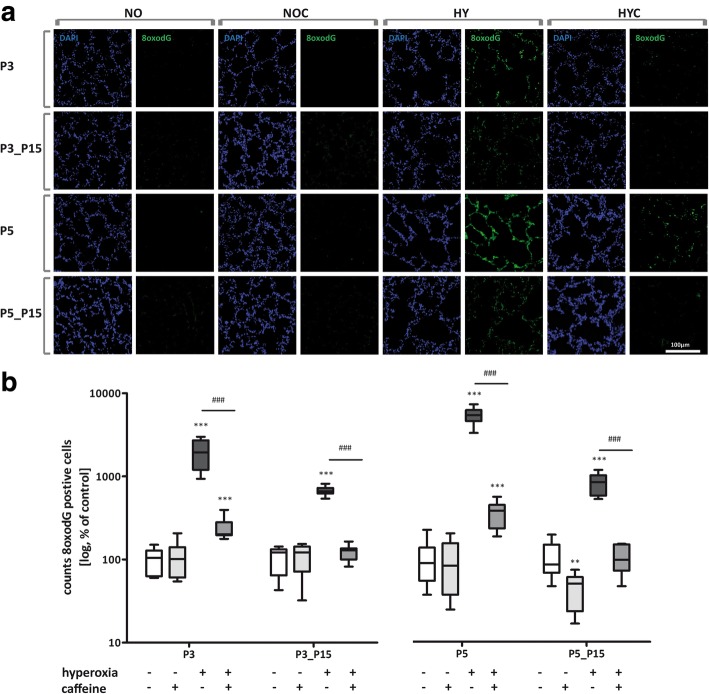
Representative microscopy images for **a**) immunohistochemical 8-oxodG staining to detect oxidative DNA damage in pulmonary sections of hyperoxia and caffeine exposed rats. Quantitation of 8-oxodG cells counts in lung sections at **b**) P3 and with recovery on P15 (P3_P15) and P5 and with recovery on P15 (P5_P15), respectively. Data are expressed relative to the normoxia-exposed control group (100%) and the 100% values are 13.3 (P3), 7.0 (P3_P15), 6.2 (P5), and 7.6 (P5_P15) cells per mm^− 2^, respectively. Error bars represent SEM, *n* = 6–8/group. **p < 0.01 and ***p < 0.001 versus control (NO); ###p < 0.001 versus hyperoxia group (HY) with Mann Whitney test

Exposure of newborn rats for 3 and 5 days with high oxygen concentrations increased the fluorescent labeling of the oxidative stress marker, which persisted during the subsequent recovery time in room air until P15 (Fig. 3 a).

Caffeine reduced the oxidative DNA damage, as reflected in the quantitation analysis. As a result of the acute hyperoxia at P3 and P5, significant DNA damage was found due to oxidative stress. Even after a recovery in room air (P3_P15 and P5_P15), the damage was still detectable. Caffeine demonstrated a strong anti-oxidative effect through significant reduction of the 8-oxodG positive cell counts during the acute phase (P3 and P5) of hyperoxia down to the levels of control litters always kept in room air (Fig. 3 b).

### Caffeine reduces the oxidative stress response

**Fig. 4:**
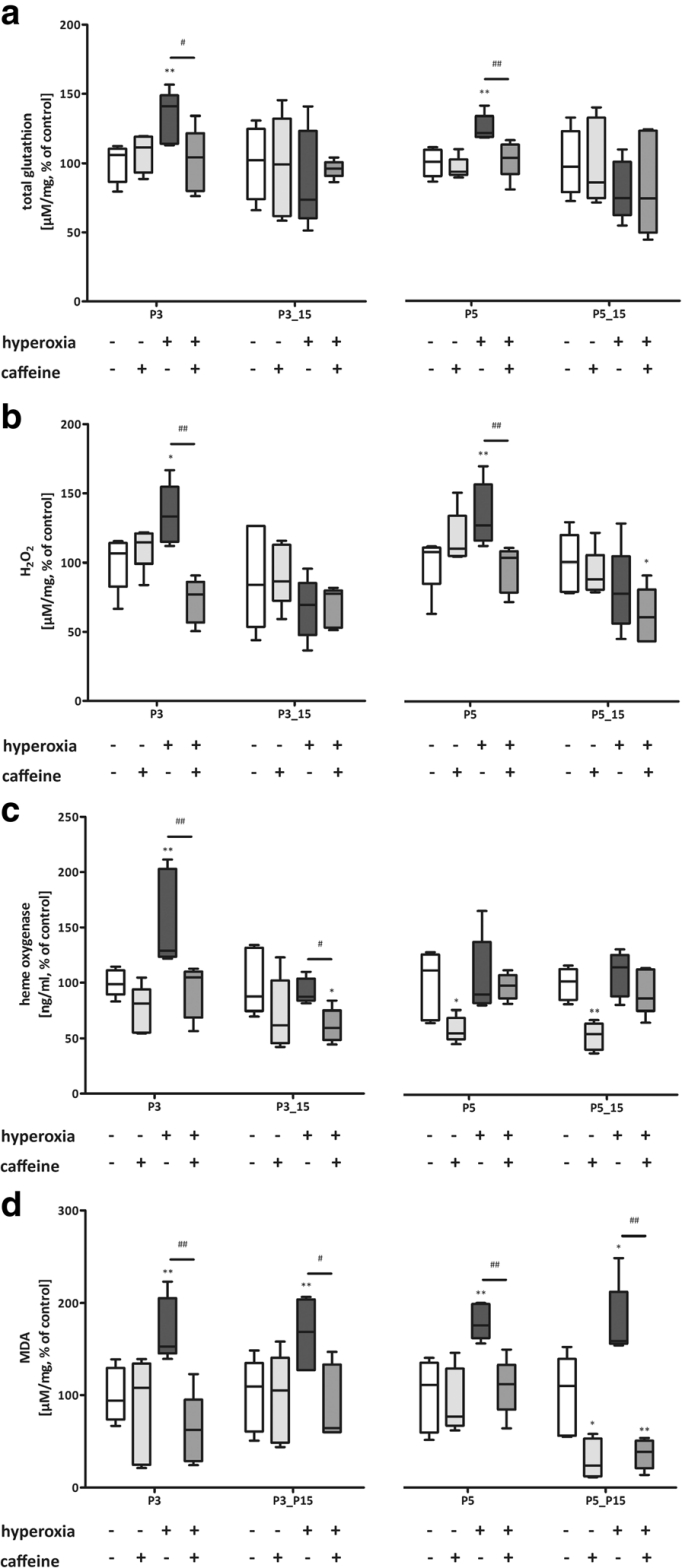
Acute hyperoxia resulted in an adequate oxidative stress response and caffeine reduced the response. Quantitation of lung homogenates by ELISA of **a**) total glutathione, **b**) H_2_O_2_, **c**) HO-1, and **d**) MDA/ lipid peroxidation with 3 days’ postnatal oxygen exposure (P3) and recovery (P3_P15) and 5 days’ postnatal oxygen exposure (P5) and recovery (P5_P15), respectively. Data are expressed relative to the normoxia-exposed control group (100%) and the 100% values are a) 19.2 μM/mg, 13.4 μM/mg, 17.8 μM/mg, and 13.7 μM/mg protein, b) 1.8 μM/mg, 3.1 μM/mg, 1.0 μM/mg, and 3.0 μM/mg protein, c) 17.2 ng/mg, 5.9 ng/mg, 20.0 ng/mg, and 5.2 ng/mg protein, and d) 3.3 μM/mg, 3.7 μM/mg, 1.3 μM/mg, and 3.3 μM/mg protein for P3 and P3_P15 or P5 and P5_P15 groups, respectively. Error bars represent SEM, *n* = 5/group. *p < 0.05 and **p < 0.01 versus control (NO); #p < 0.05 and ##p < 0.01 versus hyperoxia group (HY) with Mann Whitney test

**Fig. 5:**
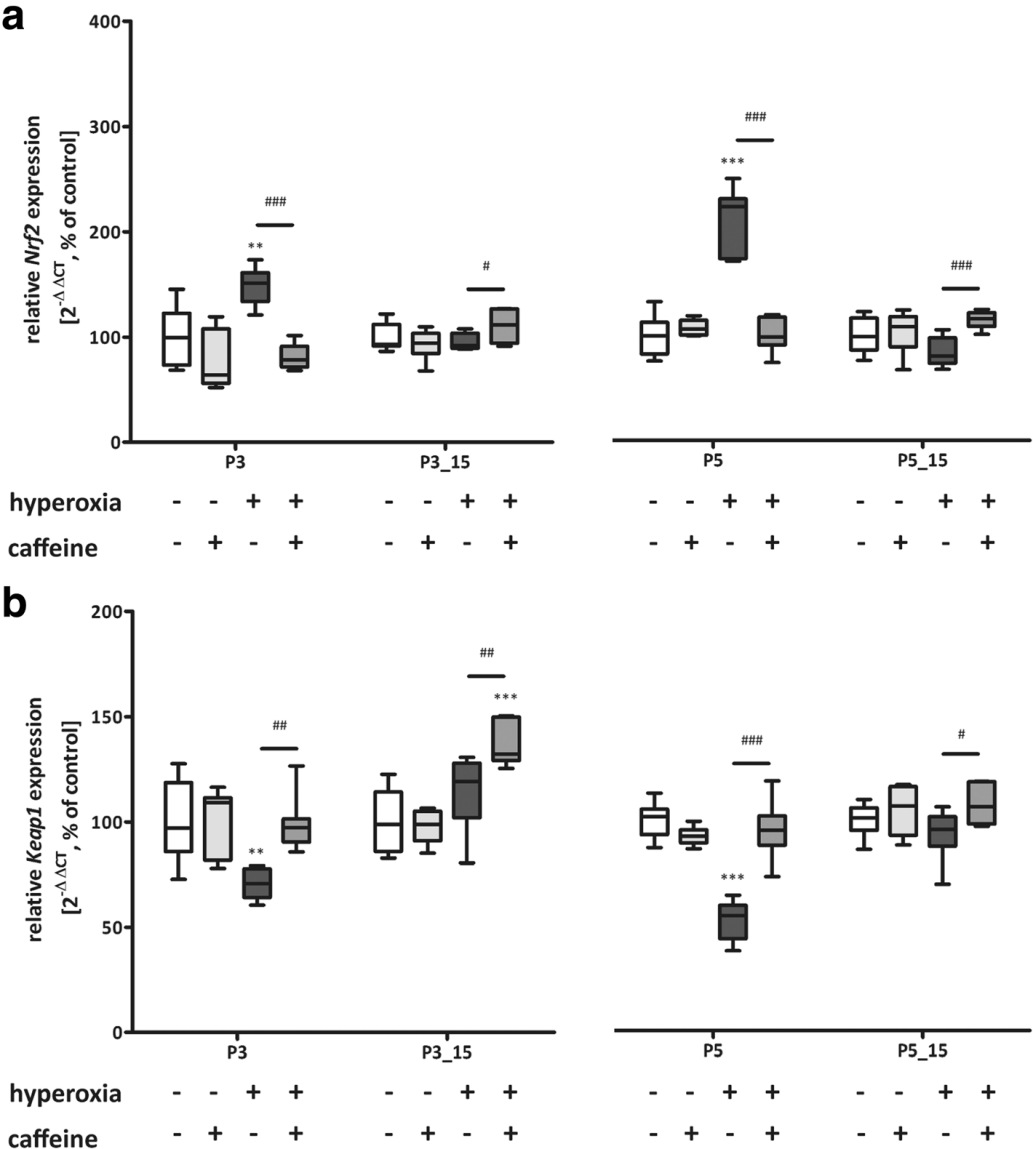
Acute hyperoxia resulted in an adequate oxidative stress response and caffeine reduced the response. Quantitation of lung homogenates by qPCR of **a**) *Nrf2*, and **b**) *Keap1* for 3 days’ postnatal oxygen exposure (P3) and recovery (P3_P15), and 5 days’ postnatal oxygen exposure (P5) and recovery (P5_P15), respectively. Data are expressed relative to the normoxia-exposed control group (100%). Error bars represent SEM, *n* = 7–8/group. *p < 0.05, **p < 0.01, and ***p < 0.001 versus control (NO); #p < 0.05, ##p < 0.01, and ###p < 0.001 versus hyperoxia group (HY) with Mann Whitney test

Hyperoxia induced an oxidative stress response at P3 and P5 for total glutathione, HO-1, H_2_O_2_, and additionally for lipid peroxidation at P5_P15 (Fig. 4 a-d). The redox-sensitive transcription factor *Nrf2* was increased while the repressor protein *Keap1* was reduced at P3 and P5, respectively (Fig. 5 a and b). Notably, caffeine acts as a potent anti-oxidative mediator and fully abolishes the oxidative stress response in hyperoxia animals at all time-points analyzed (Figs. 4 and 5).

**Fig. 6:**
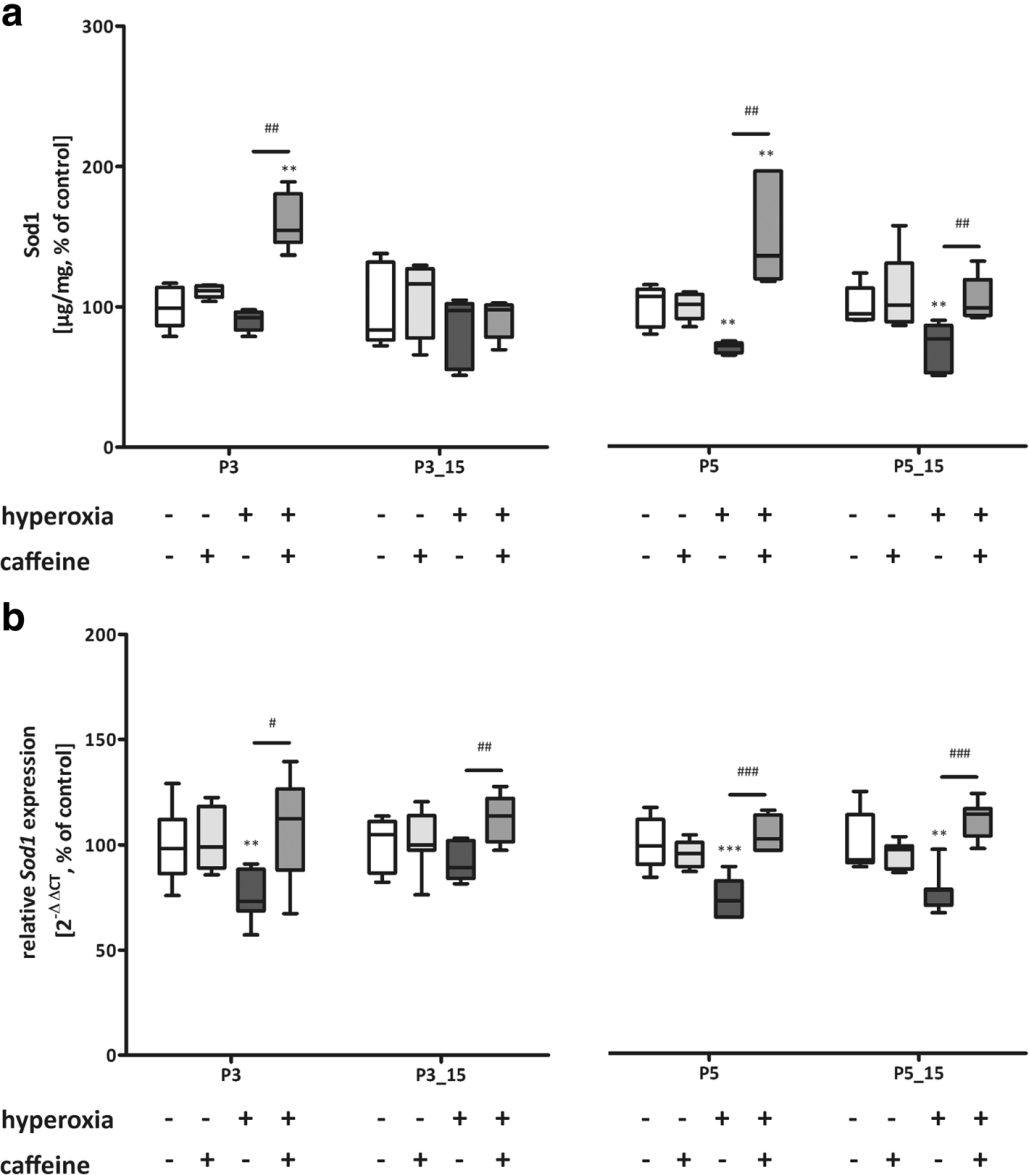
Modulation of superoxide dismutase (Sod) 1 expression through hyperoxia and caffeine. Quantitation of lung homogenates by **a**) ELISA and **b**) qPCR of for 3 days’ postnatal oxygen exposure (P3) and recovery (P3_P15), and 5 days’ postnatal oxygen exposure (P5) and recovery (P5_P15), respectively. Data are expressed relative to the normoxia-exposed control group (100%) and the 100% values for ELISA quantitation are 1.5 μg/mg, 1.0 μg/mg, 0.8 μg/mg, and 1.3 μg/mg protein for P3 and P3_P15 or P5 and P5_P15 groups, respectively. Error bars represent SEM, n = 5 (ELISA) or n = 7–8 (pPCR)/group. **p < 0.01 and ***p < 0.001 versus control (NO); #p < 0.05, ##p < 0.01, and ###p < 0.001 versus hyperoxia group (HY) with Mann Whitney test

**Fig. 7:**
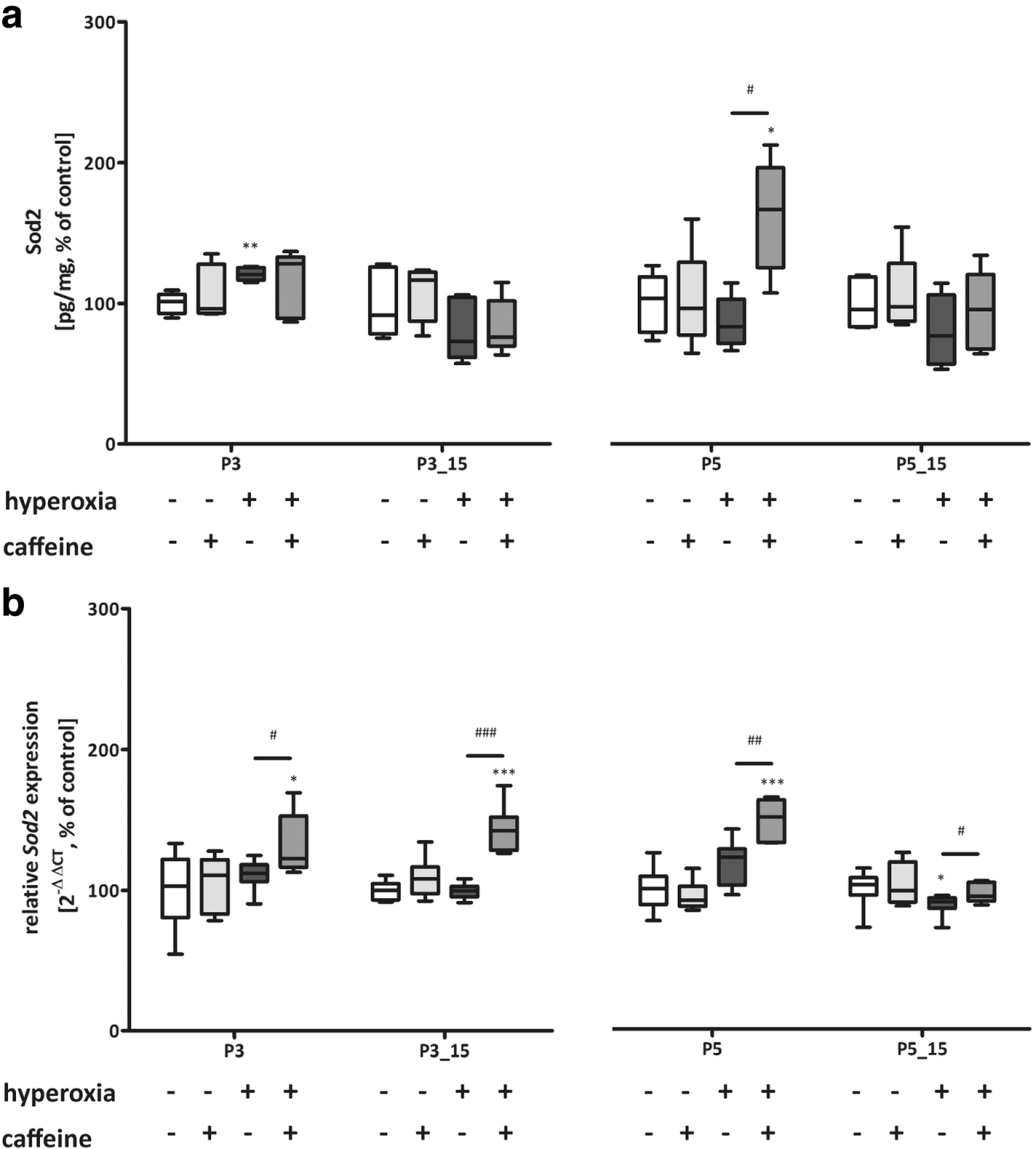
Modulation of superoxide dismutase (Sod) 2 expression through hyperoxia and caffeine. Quantitation of lung homogenates by **a**) ELISA and **b**) qPCR of for 3 days’ postnatal oxygen exposure (P3) and recovery (P3_P15), and 5 days’ postnatal oxygen exposure (P5) and recovery (P5_P15), respectively. Data are expressed relative to the normoxia-exposed control group (100%) and the 100% values for ELISA quantitation are 97.7 pg/mg, 70.4 pg/mg, 78.3 pg/mg, and 72.9 pg/mg protein for P3 and P3_P15 or P5 and P5_P15 groups, respectively. Error bars represent SEM, n = 5 (ELISA) or n = 7–8 (pPCR)/group. *p < 0.05, **p < 0.01, and ***p < 0.001 versus control (NO); #*p* < 0.05, ##*p* < 0.01, and ###*p* < 0.001 versus hyperoxia group (HY) with Mann Whitney test

**Fig. 8:**
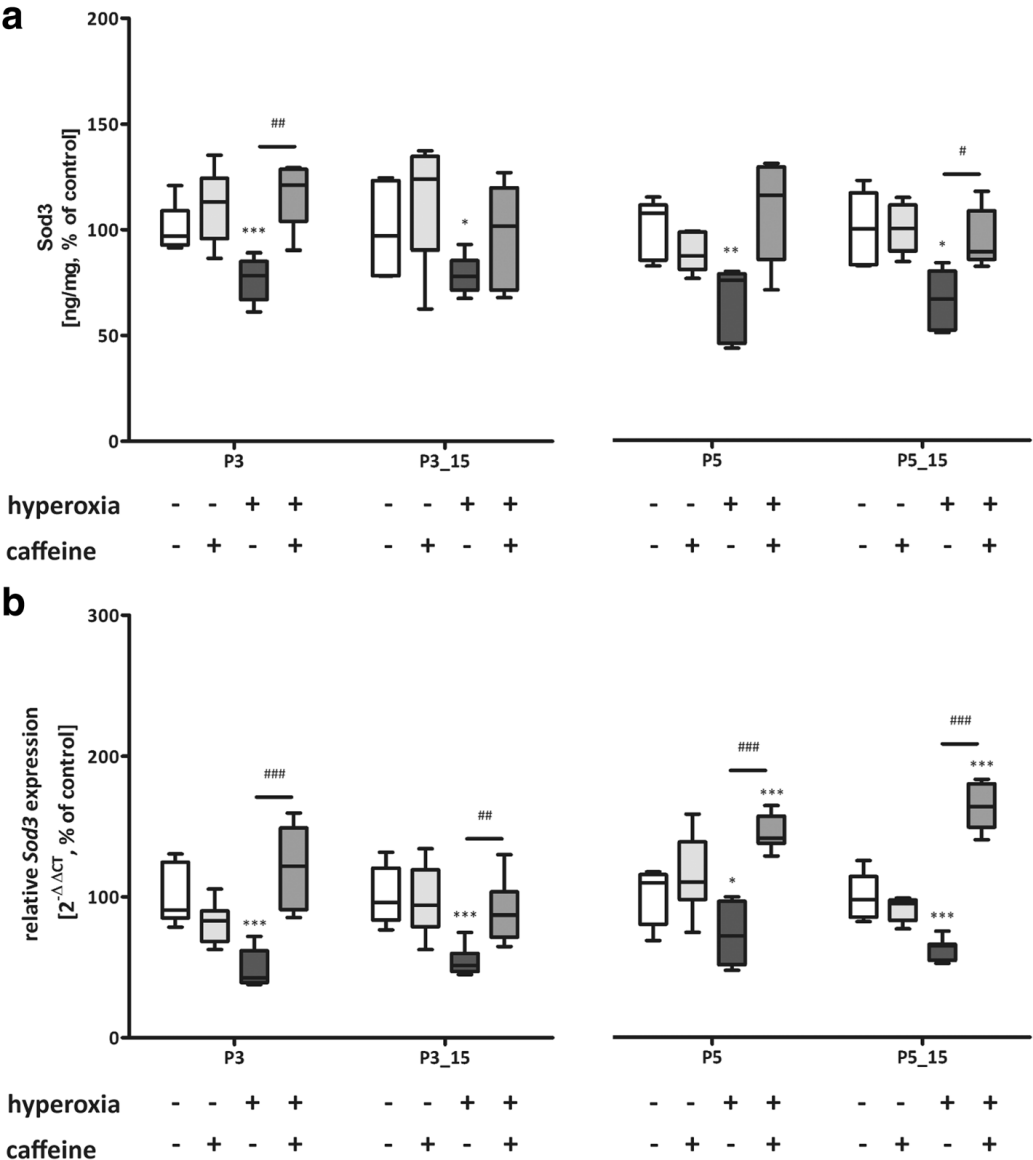
Modulation of superoxide dismutase (Sod) 3 expression through hyperoxia and caffeine. Quantitation of lung homogenates by **a**) ELISA and **b**) qPCR of for 3 days’ postnatal oxygen exposure (P3) and recovery (P3_P15), and 5 days’ postnatal oxygen exposure (P5) and recovery (P5_P15), respectively. Data are expressed relative to the normoxia-exposed control group (100%) and the 100% values for ELISA quantitation are 8.5 ng/mg, 7.2 ng/mg, 8.8 ng/mg, and 9.4 ng/mg protein for P3 and P3_P15 or P5 and P5_P15 groups, respectively. Error bars represent SEM, *n* = 5 (ELISA) or *n* = 7–8 (pPCR)/group. *p < 0.05, **p < 0.01, and ***p < 0.001 versus control (NO); #*p* < 0.05, ##*p* < 0.01, and ###*p* < 0.001 versus hyperoxia group (HY) with Mann Whitney test

Hyperoxia primarily reduced expression of Sod1 (Fig. 6 a and b) and Sod3 (Fig. 8 a and b) after 3 (P3) and/or 5 days (P5) of exposure and recovery (P3_P15, P5_P15) and of Sod2 (Fig. 7 a and b) for both exposure times with (P3_P15, P5_P15) and without (P3, P5) recovery at RNA levels. Treatment with caffeine prevented the oxidative debt from manifesting itself in changed Sod levels (Fig. 6-8).

## Discussion

Caffeine is the most common methylxanthine universally used in current neonatal practice. Its efficacy and tolerability with a wide therapeutic index and high safety have made it the drug of choice for respiratory instabilities and it is often used for many weeks with potential catabolic effects that could impact the initial weight gain of preterms. The effect of caffeine on weight loss of caffeine-treated newborn rats according to our in vivo results matches observations made in preterm infants [22, 40, 41]. This can be explained by the increased diuresis in kidneys caused by caffeine. It can only be speculated whether the amount of weight loss affects the health status of the animals investigated or of human patients being treated with caffeine. However, since this is of a temporary nature, and all treated pups after some time returned to the range of body weight similar to untreated controls, the impact of weight loss (or water loss) seems negligible and in fact could easily be treated in the context of patient medical care. From clinical studies it is known that prolonged caffeine insult may lead to a reduction in weight gain, due to a higher energy expenditure and higher oxygen consumption of the organism [42], and influenced by caffeine concentration [41].

Early oxygen exposure is one of the most important factors implicated in the development of BPD [43]. Experimental hyperoxia-induced lung injury mimics the hallmarks of human BPD [18, 27, 28], which are characterized by the damaging effect of high oxygen concentrations and the resulting structural changes, such as alveolar simplification and thickening of the alveolar septum [44–46]. Postnatal hyperoxia led to structural changes in the immature lung at exposure times over the first few days of life (P3 and P5), furthermore the damage persisted even after recovery under normoxic conditions until the transition from the saccular to the alveolar phase of lung development. Caffeine reduced this structural damage, as shown in a previous study of acute hyperoxia [47, 48].

The primary goal of the current study was the investigation of the antioxidative capacity of caffeine in a hyperoxic-based rat model of BDP, which clearly demonstrated the effective prevention of oxidative DNA damage and oxidative stress responses. While the early postnatal exposure to oxygen induced long-lasting oxidative damage to DNA and lipids after recovery in room air, no such damage was found in hyperoxic animals if they were treated with caffeine.

It is important for this animal study on hyperoxia-induced injury in the developing lung that the relevant time points of pulmonary developmental lay within the saccular phase, after the termination of the saccular phase, as well as with a recovery period after completion of the alveolar phase. The human saccular phase terminates between gestation weeks 24 and 38 (corresponding to rats P0 to P4), which correlates with very low birth weight (VLBW) infants, and the alveolar phase overlaps at about 36 weeks (corresponding to rats’ P5 to P15). Thus, our model with hyperoxia from day-of-birth to postnatal day 3 (P3) and day 5 (P5) and recovery time to day 15 (P15) provides an adequate model of lung injury to very prematurely born infants and allows studies of hyperoxic or caffeine-relevant modulations at different stages of lung development. The oxygen exposure in the first 3 and 5 days of life in rats can be regarded as equivalent to 3 to 5 weeks in humans [27]. Oxidative stress due to the transition from intrauterine hypoxia to extra-uterine hyperoxia, the need for mechanical ventilation and additional oxygen, and birth during the saccular phase of lung development are the major risk factors for respiratory distress in preterm infants, in addition to the incomplete detoxification response to free radicals [14]. Our data demonstrated clear antioxidant actions of caffeine that effectively prevent oxidative damage in the developing lung even at the high levels of oxygen that might need to be applied in preterm infants suffering from lung disease.

Hyperoxia leads to excessive generation of ROS, included hydrogen peroxide (H_2_O_2_) and hydroxyl radicals, and causes oxidative stress. The antioxidative network includes direct responses, like radical scavengers or chemical modifications, and indirect responses with up-regulation of detoxifying (e.g. HO-1) and antioxidant enzymes (e.g. superoxide dismutase) through activation of the redox-sensitive Nrf2/Keap1 pathway [49]. Adequate mechanisms are detectable in ROS-induced injury in the immature postnatal developing pulmonary system of the newborn rodent. Antioxidant therapies would be a conceivable remedy to ameliorate this process with the pathological consequences of oxidative stress.

Antioxidant properties of caffeine are highlighted by various in vivo and in vitro models [39, 50–52] and are supported by our findings. Caffeine administration caused full blocking of hyperoxia-induced oxidative stress and long-term oxidation of DNA in the lungs, both during the acute oxygen exposure and also after recovery in room air. Hyperoxia can lead in the lungs to base damage, mostly a primary effect of ROS on the DNA, but also to DNA strand breaks, due to more secondary effects of nucleases during cell death processes. DNA strand breaks primarily affect proliferating cells and type II alveolar epithelial cells [53]. The oxidative degradation and breakage of lipids can lead to changes of membrane permeability and fluidity and hence impair cell integrity, and may trigger regulated cell death [54].

High oxygen exposure increased lipid peroxidation directly after the insult and was also significantly detectable after 5-day exposure and tendentially detectable after 3-day exposure at P15, while caffeine prevented lipid peroxidation. Caffeine has also been shown to exert antioxidant properties against ROS in rat liver microsomes [51]. Tiwari et al. demonstrated an inhibition of H_2_O_2_ generation through caffeine in a hyperoxic in vitro model of human lung epithelial cells [52]. Similar effects of caffeine were detected in our hyperoxia model at P3 and P5. Glutathione itself is an essential endogenous antioxidant and directly interacts with free oxygen radicals and protects cells from ROS. The level of total glutathione in the newborn rat lung increased in response to hyperoxic exposure at P3 and P5, and caffeine counteracted this. In addition, there was a significant induction of HO-1 at P3. Li et al. demonstrated an induction of HO-1 after hyperoxic exposure for 3 days in newborn mice [55].

These results suggest that the generation of H_2_O_2_, and the increases in glutathione and of the detoxifying enzyme HO-1 are a direct effect of oxygen exposure in the lungs and that the increase of antioxidant response is intrinsic to the lung cell itself. The Nrf2/Keap1 pathway mediates the antioxidant gene regulation in response to oxidative stress, and Nrf2 is a cytoprotective mediator regulating the expression of genes coding for antioxidant, anti-inflammatory, and detoxifying proteins [56]. Oxidative stress in a murine BPD model using postnatal hyperoxia (P0 to P4) followed by short-term recovery (14 d) in normoxic conditions was associated with significantly increased mRNA expression for antioxidant genes (e.g. HO-1 and Sod) mediated by Nrf2 [57]. The redox-sensitive transcription factor *Nrf2* was induced by hyperoxia at P3 and P5, while *Keap1* was reduced. Caffeine regulated RNA expression inversely. Sod1 and Sod2 are regulated by Nrf2 [58], whereas Sod3 is a Nrf2-independent antioxidant [57] and an extracellular enzyme, tightly regulated in developing lung [59]. In our investigations, we were able to show that Sod3 was significantly reduced at mRNA and protein level at P3 and P5, and also after recovery in room air. For Sod1, reduced levels were mainly detectable after 5 days of oxygen and this persisted until postnatal day 15. The Sod2 expression was only slightly affected by oxygen. However, for all investigated superoxide dismutases, caffeine significantly enhanced expression at the RNA level and abolished the oxygen-induced deficits at the protein level for Sod1 and Sod3. Diminishment of superoxide dismutase was also described in a murine BPD model [57], in lung homogenates of ventilated juvenile pigs [60], and in a hyperoxic-induced lung injury model of adult male rats [61]. Decreased level of superoxide dismutase may be caused by increased oxygen radical formation and depletion of superoxide dismutase in reaction with significant induction of ROS [62].

With a 14-day daily intake of caffeine among adult male Wistar rats, there was a significant reduction of lipid peroxidation [63]. Caravan and colleagues demonstrated in adult female rats that sub-chronic administration of caffeine decreased the lipid peroxidation and improved the antioxidant defense in the blood and brain [64]. Teng et al. demonstrated a significant reduction of DNA oxidation with caffeine in rat pups raised in hyperoxia from P1 to P10 and recovery in room air until P21 [35].

In summary, caffeine shows established clinical effects [22, 65, 66] with high efficacy and is one of the most widely used pharmacologic agents in the neonatal intensive care unit. Recent studies have provided new insights into the mechanisms by which the reduction of respiratory distress symptoms per se or, as shown in *in vivo* and *in vitro* studies, the anti-inflammatory or antioxidant effects of caffeine may be causative [47, 52, 67, 68].

## Limitations

Some limitations of this study are to be mentioned. Predominant injurious stimuli for BPD models are hyperoxia and mechanical ventilation (reviewed in [26]). Our model used hyperoxia to induce oxidative stress with a focus on the antioxidant properties of caffeine. No statements can be made on quantitation of the structural changes, as other perfusion techniques would need to be used according to the recommended standard [69]. Paraformaldehyde is a good general-purpose fixative for immunohistochemistry and immunocytochemistry, because it does not completely destroy protein immunogenicity [70]. However, paraformaldehyde does not adequately stabilize tissue structure; the fixed lung is subject to significant mechanical distortion and collapse and pulmonary cell structures are not adequately fixed. Fixation with glutaraldehyde and is more suitable for morphological quantitative analysis and aims to preserve mainly lung volume in a defined inflation state, architectural integrity of lung parenchyma, airways, and vessels, ultrastructure of lung cells, organelles, and matrix [69]. Structural and morphological changes aspects in unity with the cellular mechanisms are currently being further investigated in this project.

Caffeine was administered every 48 h, and not in a once-daily regimen, in order to avoid elevated doses of caffeine outside of the therapeutic range that might induce pro-inflammatory responses [70]. Caffeine per se induces the inflammatory cascade and cytokine levels were highest in the time window after the application. Chavez Valdez et al. could show this clinically as well [70, 71]. Caffeine has been shown to be anti-inflammatory only in a therapeutic plasma level. The measured caffeine plasma levels were shown to be in the therapeutically comparable range of 5,5–23,7 mg/L [21] two days after administration. We deliberately opted for non-daily application to avoid the daily inflammatory insults for the rats, but also to reduce the stress on the juveniles and the dams.

## Conclusion

Caffeine treatment modulated the antioxidative response in hyperoxic-induced lung injury and suggested that caffeine acts as a potent antioxidant. Effective radical scavenging properties of caffeine could have an essential and crucial function in the treatment of oxidative stress-induced respiratory diseases. Finally, the pathogenesis of neonatal respiratory distress syndrome or BPD involves the formation of ROS and may be amenable to reductions in exposure to oxygen as well as treatment with antioxidants [17, 22, 66, 72].

## Additional files

Additional file 1: **Figure S1.** Representative haematoxylin and eosin (H&E)-stained sections of uninjured and oxygen-injured animals at all time-points with and without caffeine application. (TIF 6680 kb)

Additional file 2: **Table S1.** Sequences of oligonucleotides. (DOCX 13 kb)

## Abbreviations

8-oxodG: 8-oxo-2′-deoxyguanosine
BPD: Bronchopulmonary dysplasia
FAM: 6-carboxyfluorescein
HO-1: Heme oxygenase-1
Hprt: Hypoxanthine-guanine phosphoribosyl-transferase
Keap1: Kelch-like ECH-associated protein 1
MDA: Malondialdehyde
Nrf2: NFE2-related factor 2
P: Postnatal day
PBS: Phosphate buffered saline
ROS: Reactive oxygen species
ROX: 6*-*carboxy*-*X*-*rhodamine
SOD/Sod: Superoxide dismutase
TAMRA: Tetramethylrhodamine
TBARS: Thiobarbituric acid reactive substances
