# Supplementary figures and images for: Antioxidative effects of caffeine in a hyperoxia-based rat model of bronchopulmonary dysplasia
Source: Respir Res. 2019 May 10;20:88. doi: 10.1186/s12931-019-1063-5 (PMC6511176; doi:10.1186/s12931-019-1063-5)

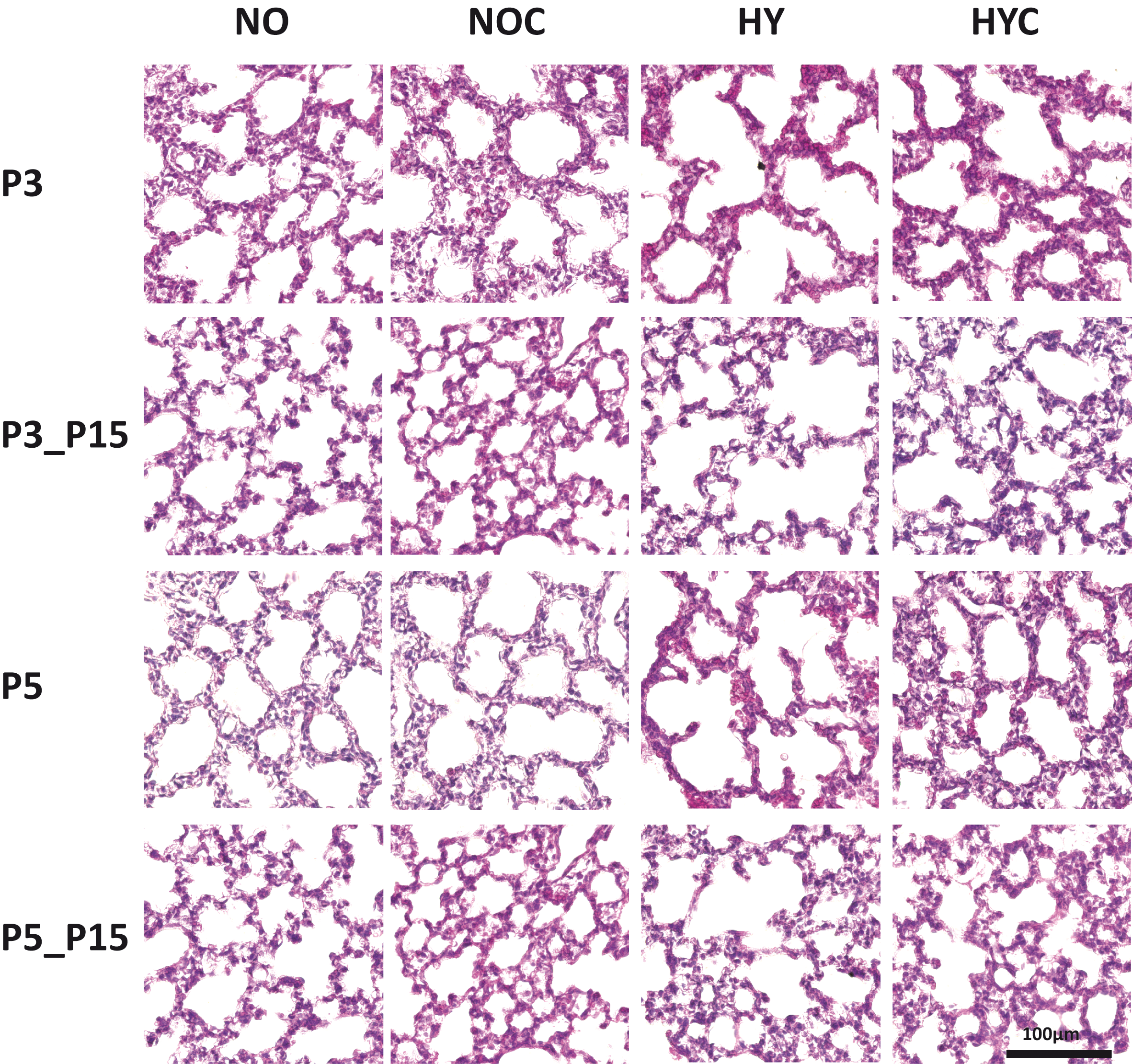

Supplement: Supplementary file 1 — Figure S1. Representative haematoxylin and eosin (H&E)-stained sections of uninjured and oxygen-injured animals at all time-points with and without caffeine application. (TIF 6680 kb) [file 12931_2019_1063_MOESM1_ESM.tif]
