# Supplementary material for: Antioxidative effects of caffeine in a hyperoxia-based rat model of bronchopulmonary dysplasia
Source: Respir Res. 2019 May 10;20:88. doi: 10.1186/s12931-019-1063-5 (PMC6511176; doi:10.1186/s12931-019-1063-5)
Supplement: Supplementary file 2 — Table S1. Sequences of oligonucleotides. (DOCX 13 kb) [file 12931_2019_1063_MOESM2_ESM.docx]

**Table 1** Sequences of oligonucleotides

| **cDNA** | **oligonucleotide sequence 5´-3´** | **Accession No.** |
| --- | --- | --- |
| ***Hprt*** | | |
| forward | GGAAAGAACGTCTTGATTGTTGAA | NM_012583.2 |
| reverse | CCAACACTTCGAGAGGTCCTTTT |  |
| probe | FAM-CTTTCCTTGGTCAAGCAGTACAGCCCC-TAMRA |  |
| ***Keap1*** | | |
| forward | GATCGGCTGCACGGAACT | NM_057152.2 |
| reverse | GCAGTGTGACAGGTTGAAGAACTC |  |
| probe | FAM-CTCGGGAGTATATCTACATGC-TAMRA |  |
| ***Nrf2*** | | |
| forward | ACTCCCAGGTTGCCCACAT | NM_031789.2 |
| reverse | GCGACTCATGGTCATCTACAAATG |  |
| probe | FAM-CTTTGAAGACTGTATGCAGC-TAMRA |  |
| ***Sod1*** | | |
| forward | CAGAAGGCAAGCGGTGAAC | NM_017050.1 |
| reverse | CCCCATATTGATGGACATGGA |  |
| probe | FAM-TACAGGATTAACTGAAGGCG-TAMRA |  |
| ***Sod2*** | | |
| forward | GACCTACGTGAACAATCTGAACGT | NM_017051.2 |
| reverse | AGGCTGAAGAGCAACCTGAGTT |  |
| probe | FAM-ACCGAGGAGAAGTACCACGA-TAMRA |  |
| ***Sod3*** | | |
| forward | GGAGAGTCCGGTGTCGACTTAG | NM_012880.1 |
| reverse | CTCCATCCAGATCTCCAGGTCTT |  |
| probe | FAM-CTGGTTGAGAAGATAGGCGA-TAMRA |  |

*Hprt,* hypoxanthine-guanine phosphoribosyl-transferase; Keap1, *Kelch-like ECH-associated protein 1;* Nrf2*,* *NFE2-related factor 2;* Sod*, superoxide dismutase*
